# Editorial: Enhancing the social skills and social competence for children and adolescents with autism spectrum disorder

**DOI:** 10.3389/fpsyt.2026.1844363

**Published:** 2026-05-29

**Authors:** Magdalena Budisteanu

**Affiliations:** 1 Psychiatry Research Laboratory, Prof. Dr. Alex. Obregia Clinical Hospital of Psychiatry, Bucharest, Romania; 2 Medical Genetics Laboratory, Victor Babes National Institute of Pathology, Bucharest, Romania; 3 Faculty of Medicine, Titu Maiorescu University, Bucharest, Romania

**Keywords:** autism, environmental contribution, intervention, neurobiology, research

## Executive summary

Autism spectrum disorder (ASD) is defined as a common neurodevelopmental disorder characterized by long-term difficulties in social interaction and communication, along with stereotypical behavior. This Research Topic, “Improving social skills and social competence in children and adolescents with Autism Spectrum Disorder,” summarizes the contribution of ten scientific papers that approach original intervention strategies, the biological and environmental effects on social competence, and the lived experiences of caregivers.

The body of work discussed in this editorial points to meaningful progress in the development and application of evidence-based approaches. Among the most notable outcomes are the demonstrated effectiveness of adapted social skills interventions within public mental health services, the emerging promise of biomedical techniques such as low-intensity transcranial focused ultrasound, and the positive secondary effects of therapeutic horsemanship on caregiver well-being. In addition, investigations examining narrative competencies in first-degree relatives, the influence of circadian rhythm disruption—particularly social jet lag—on core symptom expression, and structural alterations within reward-related neural circuits contribute to a more nuanced understanding of ASD as a complex and multidimensional condition.

Important methodological advances are also reflected in the validation of assessment instruments adapted for telehealth delivery, as well as in the development of user-oriented museum design approaches, both of which highlight the growing emphasis on accessibility and inclusive spaces. Moreover, qualitative findings exploring the emotional burden experienced by caregivers—particularly within specific cultural settings such as China—draw attention to the necessity of comprehensive and culturally sensitive support frameworks. Collectively, these studies help shape emerging research priorities and inform clinical strategies focused on enhancing social participation and overall quality of life for individuals with ASD.

## 1 Introduction

Autism Spectrum Disorder (ASD) is characterized by early-onset differences in social communication and interaction that frequently continue across the lifespan. These challenges may influence social reciprocity, pragmatic language, adaptive functioning, and overall well-being. When not addressed through appropriate intervention, social difficulties can intensify over time, potentially limiting community participation and interpersonal development.

Recent epidemiological reports indicate that ASD prevalence continues to rise. According to 2023 data from the Centers for Disease Control and Prevention (CDC), approximately 1 in 36 eight-year-old children in the United States has been identified with autism, reflecting a substantial increase compared to earlier estimates. Comparable data from China suggest a prevalence rate of roughly 0.7% among children aged 6 to 12 years.

This Research Topic seeks to advance understanding of how social development unfolds in individuals with ASD and to examine intervention models tailored to individual profiles. The included studies investigate cognitive, neural, and behavioral dimensions of autism, while also addressing contextual factors such as family engagement, sensory processing differences, and co-occurring emotional conditions including anxiety and depression. By connecting empirical findings with applied practice, the Research Topic contributes to a broader conceptual framework for strengthening social competence in autistic youth.

## 2 Emerging and innovative intervention approaches

A prominent focus of this Research Topic involves structured programs designed to foster social functioning. These approaches range from structured psychosocial programs to emerging biomedical techniques, reflecting the growing diversity of intervention strategies in the field. **Table 1** summarizes selected studies on ASD interventions and research approaches, including the type of intervention, methodological category, target population, and key findings or outcomes reported by each study.

**Table 1:** Summary of interventions and research approaches in autism spectrum disorder (ASD).

Study	Intervention/approach	Category	Population	Key findings
Mairena et al.	SAEIP (Social Adjustment Enhancement Intervention Program)	Psychosocial/Group-based	Children & Adolescents (8–17 years)	Significant improvements in eye contact and functional communication, particularly in younger children with higher verbal IQ
Qian et al.	Kano-QFD-PUGH Based Museum Design	Environmental/Design	Children with ASD	Identification of “Science and Technology Interactive” designs (AR/VR) that increased user satisfaction by ~23%
Landau et al.	Visual Scaffolding in Narrative	Cognitive/Scaffolding	ASD individuals & first-degree relatives	Visual aids support storytelling; narrative quality subtly impacted in relatives (BAP)
Cheng et al.	Low-intensity Transcranial Focused Ultrasound (tFUS)	Biomedical/Neuromodulation	1 Boy with ASD (Case Study)	Improved social interaction and stereotypical behavior; increased functional connectivity in brain regions
Barron et al.	Therapeutic Horsemanship (TH)	Animal-Assisted	Caregiver-Child dyads	Statistically significant reduction in caregiver stress (DASS-21 scores) over a 16-week semester
Fan and Ko	Qualitative Parenting Support	Qualitative/Cultural	Chinese Caregivers	Explores emotional phases and cultural “hope for ordinariness” to inform support systems
Chen et al.	Circadian Rhythm Management (Lifestyle)	Environmental/Behavioral	Children (2–7 years)	Correlation between “social jet lag” and symptom severity; emphasizes regular routines
Traetta et al.	e-ABaCo (Telehealth Assessment)	Technological/Assessment	Autistic adolescents	Validated equivalence between remote and face-to-face assessment of pragmatic communication skills
Qiao et al.	Knowledge Mapping (CiteSpace)	Bibliometric/Review	Academic Research Community	Visualized shift in ASD research toward environmental, hormonal, and diverse population factors
Sun et al.	MRI Feature Refinement (Diagnostic)	Mechanistic/AI	Children & Adolescents (7–18 years)	Identified structural abnormalities in reward system linked to social motivation deficits

### 2.1 Adapted social skills interventions in public mental health services

Mairena et al. conducted a randomized, waitlist-controlled pilot trial evaluating an adapted Social Adjustment Enhancement Intervention Program (SAEIP). Implemented within a university-affiliated public mental health hospital, the project aimed to demonstrate the feasibility of delivering structured, evidence-informed services within publicly funded systems.

Seventy-nine autistic participants between 8 and 17 years old completed the 10-session group intervention. Social behavior changes were measured using structured observational methods, while parent-report questionnaires assessed internalizing symptoms.

The results indicated meaningful gains in eye contact and functional communication among younger participants and those with higher verbal intelligence. In addition, reductions in internalizing symptoms were observed within the intervention group. Although statistical significance was not achieved across the entire sample, the direction of change consistently favored the intervention. These findings underscore the importance of tailoring interventions to individual developmental and cognitive profiles while also demonstrating the feasibility of implementing such programs in public service settings.

### 2.2 Therapeutic horsemanship and effects on caregivers

Barron et al. examined the broader impact of therapeutic horsemanship (TH), an animal-assisted intervention, with particular attention to caregiver outcomes. While benefits for autistic children have been documented, research exploring secondary effects on caregivers remains limited.

In this 16-week pilot study, 13 caregiver-child dyads participated in structured TH sessions. A mixed-methods design combined quantitative assessment with qualitative interviews. Results demonstrated a statistically significant reduction in caregiver stress (p = 0.03) as measured by the DASS-21 scale. Interview data further revealed recurring themes related to service navigation challenges, financial strain, and limited support resources. Importantly, caregivers described reduced emotional burden as their children engaged in TH, suggesting that intervention benefits may extend to the entire family system and highlighting the importance of incorporating caregiver outcomes in intervention research.

### 2.3 Low-intensity transcranial focused ultrasound (tFUS)

Cheng et al. presented a case study exploring low-intensity transcranial focused ultrasound stimulation (tFUS) as a novel therapeutic avenue. Currently, no pharmacological treatment directly targets the core social and behavioral characteristics of ASD.

In this report, tFUS was administered to the left dorsolateral prefrontal cortex of a child with ASD for 30 minutes daily across four weeks (20 sessions total). Clinical rating scales and functional near-infrared spectroscopy (fNIRS) were used to monitor outcomes. Improvements were observed in social interaction and repetitive behaviors, alongside increased functional connectivity involving the primary somatosensory cortex. While these results are preliminary and based on a single case, they suggest that modulation of cortical activity may offer a promising complementary strategy for addressing core ASD features.

## 3 Neurobiological and environmental contributors to social development

Understanding ASD requires attention to both biological substrates and contextual influences, which together shape behavioral expression.

### 3.1 Brain structure and the reward system

Sun et al. applied a machine learning-based multi-stage feature refinement approach to identify structural brain characteristics distinguishing individuals with ASD from typically developing peers. The study included 175 autistic participants and 69 controls aged 7–18 years.

Nine structural features were identified as particularly discriminative, many associated with neural circuits involved in reward processing, including hippocampal subregions. Alterations within these systems may contribute to differences in social motivation and engagement, offering insight into the neurobiological underpinnings of ASD.

### 3.2 Circadian rhythm disruption and social jet lag

In addition to structural brain differences, environmental factors such as sleep patterns also play a critical role in shaping social and behavioral outcomes.

Chen et al. investigated the association between social jet lag (SJL)—a misalignment between biological rhythms and social schedules—and core autism symptoms in young children.

Nearly half of the sampled children (49.8%) experienced sleep difficulties, with marked differences between weekday and weekend sleep patterns. Among children aged 2–3 years, greater SJL was positively correlated with more pronounced core symptoms. These findings underscore the potential impact of circadian regulation on behavioral expression and highlight the importance of consistent sleep routines.

## 4 Communication and narrative competence

Social functioning is closely linked to language use, especially narrative and pragmatic skills.

### 4.1 Narrative skills and familial patterns

Landau et al. explored storytelling ability in autistic individuals and their first-degree relatives to examine possible associations with the Broad Autism Phenotype. Participants completed both visually supported storytelling (“First Telling”) and unsupported retell tasks.

Across groups, retell narratives were generally less elaborated. Notably, patterns of narrative performance overlapped between autistic participants and their relatives, suggesting subtle familial influences. The removal of visual cues appeared to amplify pragmatic challenges, mirroring conversational demands in everyday contexts.

### 4.2 Telehealth-based pragmatic assessment

Building on these findings, recent work has also explored how such communicative abilities can be reliably assessed using remote technologies.

Traetta et al. evaluated a telehealth adaptation of the Assessment Battery for Communication (e-ABaCo). Thirty autistic adolescents (15 assessed remotely, 15 in person) were compared to typically developing controls.

Results demonstrated strong equivalence between remote and face-to-face administration. Both autistic groups showed distinct pragmatic profiles relative to controls. The study supports telehealth as a valid and accessible modality for pragmatic assessment.

## 5 Inclusive design and research mapping

Research on autism is increasingly expanding beyond clinical and behavioral domains to include environmental design and large-scale knowledge synthesis. Together, these approaches contribute to a broader understanding of how social participation can be supported across different contexts.

### 5.1 Autism-friendly museum environments

Qian et al. employed structured user-centered design models to create museum environments responsive to the needs of autistic children. Using the Kano model, Quality Function Deployment, and the PUGH matrix, the researchers identified design priorities.

An interactive science and technology museum model incorporating AR/VR elements and spatial guidance yielded the highest satisfaction ratings, with approximately a 23% improvement in user satisfaction. The study illustrates how systematic design processes can enhance engagement and accessibility.

### 5.2 Trends in autistic traits research

Complementing these applied design approaches, Qiao et al. provided a broader perspective through bibliometric analysis of autistic traits research spanning 1997–2024. The review of 1,044 publications revealed steady growth in scholarly output, with significant contributions from England, the United States, and Australia.

The field has expanded beyond genetic and behavioral paradigms to incorporate environmental, hormonal, and psychiatric dimensions, reflecting increased recognition of autism’s heterogeneity. This shift reflects a growing recognition of autism as a complex and heterogeneous condition, reinforcing the need for interdisciplinary research and practice.

## 6 Caregiver perspectives in cultural context

Fan and Ko provided qualitative insight into the lived experiences of Chinese caregivers of children with ASD. Four recurring themes emerged: the enduring hope for typical development, tension between expectations and reality, progression through emotional adjustment phases, and resilience in daily caregiving.

These findings highlight the cultural shaping of parental experiences and emphasize the need for context-sensitive support services. They also underscore the importance of integrating caregiver perspectives into intervention planning, policy development, and service delivery.

## 7 Future research priorities

The collective findings point toward several important directions for future research. These priorities reflect the need for more integrative, longitudinal, and context-sensitive approaches to understanding and supporting social development in autism:

- Longitudinal evaluation of psychosocial and biomedical interventions,
- Greater emphasis on individualized, profile-based intervention planning,
- Further investigation of reward system alterations and social motivation
- Expanded family-centered outcome research,
- Cross-cultural comparative studies,

These priorities highlight the importance of advancing interdisciplinary research that bridges clinical practice, neurobiological mechanisms, and real-world contexts of care.

## 8 Conclusion

This Research Topic illustrates the inherently multidisciplinary nature of autism research. The studies span clinical trials, neurobiological investigations, remote assessment innovations, environmental design strategies, and caregiver narratives. Together, they reinforce the understanding that enhancing social competence in ASD requires coordinated attention to biological mechanisms, environmental structures, and family well-being. By refining evidence-based practices and promoting inclusive systems, this body of work contributes meaningfully to advancing participation and quality of life for individuals on the autism spectrum.

